# The importance of the Peritoneal Cancer Index (PCI) to predict surgical outcome after neoadjuvant chemotherapy in advanced ovarian cancer

**DOI:** 10.1007/s00404-022-06527-y

**Published:** 2022-03-31

**Authors:** Friederike Luise Rawert, Veronica Luengas-Würzinger, Sabrina Claßen-Gräfin von Spee, Saher Baransi, Esther Schuler, Katharina Carrizo, Anca Dizdar, Peter Mallmann, Björn Lampe

**Affiliations:** 1 Department Gynecology and Obstetrics, Florence-Nightingale-Krankenhaus, Kreuzbergstr. 79, 40489 Duesseldorf, Germany; 2 Department Gynecology and Obstetrics, Universitätsklinikum Köln, Cologne, Germany

**Keywords:** Ovarian cancer, Prognostic score, Complete cytoreductive surgery, PCI score, Peritoneal Carcinomatosis Index, Neoadjuvant chemotherapy

## Abstract

**Purpose:**

Achieving complete cytoreduction (CCR) is crucial for a patient’s prognosis with advanced epithelial ovarian cancer (EOC). So far, prognostic predictors have failed to predict surgical outcome after neoadjuvant chemotherapy (NACT). In clinical trials, scores were used to predict operability in recurrent ovarian cancer (Harter et al. in N Engl J Med 385(23):2123–2131, 2021) but there is no known prediction score for CCR after NACT. The Peritoneal Cancer Index (PCI) is an established tool to predict surgical outcome in primary setting (Lampe et al. in 25:135–144, 2015). We now examined the predictive power of the PCI to achieve CCR after NACT.

**Methods:**

In this single-center study, the data of patients with advanced stage EOC (FIGO > IIIb) treated between 01/2015 and 12/2020 were analyzed retrospectively. Inclusion criteria were a mandatory staging laparoscopy, a PCI score > 25, and NACT. CT scans were analyzed in blinded fashion according to RECIST criteria (Borgani et al. in 237; 93–99, 2019) Reaction of PCI after NACT was compared with the analysis of radiologic imaging and CA-125 levels.

**Results:**

Three hundred and sixteen patients were screened, 62 were treated with NACT, and 23 were included in our analysis. 87% of cases presented with an FIGO IIIc stadium. The reduction of PCI itself after NACT showed to be the most powerful predictor for achieving CCR. The reduction of the initial PCI score by minimum of 8.5 points was a better predictor for CCR than reaching a PCI < 25. In contrast to data deriving from patients undergoing primary debulking surgery (PDS), we found a PCI of 17, rather than 25, to be a more valuable cut-off for CCR in neoadjuvant-treated patients.

**Conclusion:**

The extend of PCI reduction after NACT is a better predictor for achieving CCR compared with CA125 levels and radiologic imaging. The PCI must be assessed differently in neoadjuvant setting than in a primary situation. CCR was most likely for a post-NACT PCI < 17.

## Introduction

Epithelial Ovarian cancer (EOC) presents at an advanced stage (> FIGO IIa) in over 75% of all cases [4, 5]. Complete Cytoreduction (CRR) is the most important prognostic treatment factor for the patients [6–10]. However, extensive peritoneal carcinomatosis is usually the limiting factor when it comes to achieving CRR. Optimal patient selection is one of the most important factors influencing the outcome of cytoreductive surgery [5, 11]. Criteria against abdominal debulking are for example the diffuse deep infiltration of the root of the small bowel mesenterium or diffuse carcinomatosis of the small bowel involving such large parts that resection would lead to short bowel syndrome [11].

In colorectal cancer, the PCI (Peritoneal Cancer Index) after Sugarbaker [12] is an established scoring system to objectify the extent of peritoneal carcinomatosis and define whether a patient is considered to be operable or not. Kroll et al. [2] were able to show that the PCI score is also applicable for primary ovarian cancer. They defined a cut-off PCI score < 25 to be favorable for CCR (in colorectal cancer, the cut-off is defined as PCI < 20 [12]). Angeles et al. [13] showed that even if the laparoscopic assessment underestimated the final PCI score in EOC by 2 points compared to laparotomy, CCR and laparoscopic PCI were significantly associated. If successful cytoreductive surgery is not expected in a patient (PCI > 25), a neoadjuvant therapy concept should be considered to reduce the tumor burden followed by an interval debulking surgery (IDS) [5, 11, 14].

To the best of our knowledge, there are no reliable prognostic markers for the surgical outcome of IDS after NACT. Even though a lot of research has been done to identify patients that benefit from upfront surgery in a primary setting [11, 15, 16], there are very little data on predictive markers for surgical outcome in a neoadjuvant setting. Radiologic imaging and CA-125 levels are widely used to monitor the disease under chemotherapy [5, 17], but so far no safe and practical method has been identified to predict the surgical outcome [18–22]. The histopathological tool “chemotherapy response score” (CRS) is used to evaluate the response of NACT in EOC, but was not superior to other controversial methods [23]. Some data suggest that radiologic imaging with a scoring system might predict surgical outcome, but is strongly depended on the reader variability [24]. Asp et al. used CT imaging to assess ascites volume to predict surgical outcome in upfront surgery [25], but did not look into the neoadjuvant setting. Other data suggested that a preoperative serum CA125 level of < 30 U/ml may be a useful predictor of achieving complete surgery, but the group did not consider CA15 levels before NACT in their analysis, and therefore, a selection bias could not be excluded [26]. A few studies looked into evaluated regression coefficients and absolute preoperative CA-125 levels and showed significant correlation with cytoreductive results [19–21]. However, they failed to define a clear cut-off value for clinical practice.

There are very little data on the predictive power of the PCI after NACT in colorectal cancer, too. Bhatt et al. [27] could recently show that a pathological complete remission was correctly predicted for 47% of the patients by imaging and for 44.4% of the patients by surgical evaluation of the PCI in colorectal cancer. This might indicate a similar predictive value of the PCI for surgical outcome of ovarian cancer patients at the time of interval debulking surgery. It would be only logical to assume that patients who underwent NACT and show a PCI score after treatment < 25 should undergo successful CCR.

If it could be shown that one of the markers or a combination of those could predict the surgical outcome with clear cut-off values, treatment strategies could derive from it and the outcome of patients could benefit.

## Patients and methods

Between 01/2015 and 12/2020, a total of 316 patients with primary ovarian, fallopian tube, or peritoneal cancer were treated in our clinic (in the following, only called “ovarian cancer”). 62 of those presented with an advanced stage (FIGO stages IIIb—IVb) and were treated with a neoadjuvant therapy according to an interdisciplinary tumorboard recommendation, when CCR could not be anticipated. All patients underwent complete staging before enrollment. This included a diagnostic laparoscopy for histological confirmation of the diagnosis and evaluation of the PCI as well as CT scans of the thorax and abdomen and tumor markers (HE4, CA125). CCR was not anticipated whenever a PCI was > 25 during laparoscopy. Patients underwent neoadjuvant platin-based chemotherapy with Carboplatin AUC 5 and Paclitaxel 175 mg/m^2^ usually every 3 weeks. After 3 cycles, patients underwent radiologic re-staging as well as re-evaluation of the tumormarker. If CCR was now anticipated, IDS was performed via laparotomy and the PCI was evaluated again intraoperatively. However, if the effect of NACT was not considered strong enough, it could be followed by another 3 cycles NACT according to the recommendation of the interdisciplinary tumorboard.

**Fig. 1:**
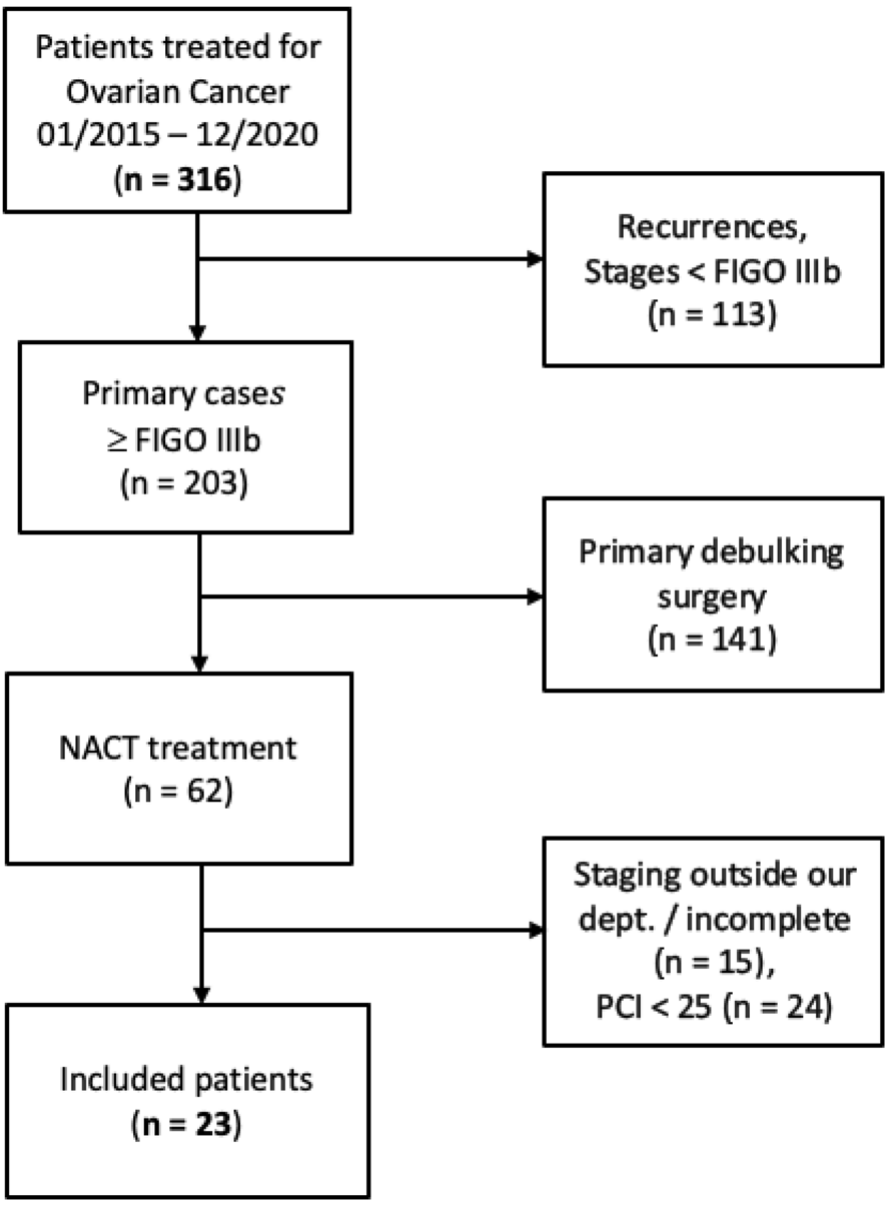
Patient screening process

Only patients with a PCI > 25 were included in our analysis, according to the data published by Kroll et al. [2]. Patients without complete preoperative staging were excluded. As were patients that did not receive a preoperative, laparoscopic assessment of the PCI score by a trained onco-gynecologist from our department. Only patients where CCR was preoperatively anticipated by the interdisciplinary tumorboard were included. The screening process is shown in Fig. 1.

Finally, 23 patients were included in our analysis according to our screening process. The patient characteristics are shown in Table 1.

The analysis was approved by the appropriate Institutional Review Board (IRB), and requirement for written informed consent was waived by the IRB.

Statistical analysis was performed using patient files, surgical records, radiological records, and intraoperative photographic documentation.

The baseline CT scans were retrospectively reviewed by one radiologist using the RECIST (Response Evaluation Criteria in Solid Tumors) 1.1 guidelines [28]. To avoid a selection bias, the radiologist blindly and randomly analyzed the CT scans of all patients without knowing the time of the study nor the patient’s name. Serum CA-125 levels before and after the neoadjuvant treatment were investigated. The PCI was evaluated at the time of laparoscopy before NACT and at the time of IDS via longitudinal incision by gyneco-oncologists.

Our primary endpoint was the accuracy of prediction for CCR of the single markers to achieve CCR during the IDS.

The data were summarized descriptively. Absolute and relative frequencies, arithmetic mean values with their standard deviations, and medians with respective quartiles were calculated. We used the binary logistic regression method to assess the quality of the chosen predictors regarding complete resection. Crosstabs were used to analyze the RECIST data. The associations were evaluated using Mann–Whitney *U* or Chi-square tests, defining a statistical significance as *p* < 0.05. We used the receiver-operating characteristic (ROC) concept to evaluate the selectivity of the sensitivity and specificity of the predictors PCI < 25 and PCI reduction. In a second analysis, we used ROC curves for the PCI after NACT and CCR. We used Youden’s Index to determine which threshold value is best suited to distinguish between two groups in a measurement using the ROC curves. Youden’s Index is defined for all points of an ROC curve, and the maximum value of the index may be used as a criterion for selecting the optimum cut-off point when a diagnostic test gives a numeric rather than a dichotomous result [29].

**Table 1:** Patient characteristics

	*n*	%
FIGO stage		
IIIb	1	4.3
IIIc	20	87
IVa	1	4.3
IVb	1	4.3
Complete cytoreduction at IDS	17	73.9

All statistical analyses were performed using the IBM SPSS^®^ Statistics software version 27 for Macintosh (IBM, Armonk, NY, USA).

## Results

Twenty-three patients were included in our analysis. 87% of them presented with an FIGO IIIc stadium. The mean age was 63 years. Patients were comparable regarding age, FIGO stage at time of diagnosis, and BMI.

Complete cytoreduction (CCR) was achieved in 73.9% of the patients after NACT. In 6 patients, CCR was not completed, due to persistent extensive peritoneal carcinomatosis at the mesenterium, which did not show in the radiologic re-staging but only during laparotomy. Tumor debulking was performed, followed by 3 cycles’ additional adjuvant chemotherapy. In the case of one patient, liver metastases were found intraoperatively, which were not classified as parenchymal preoperatively either during laparoscopy or in imaging. A resection was not possible due to the localization on the hilum.

The statistical results of our patient cohort are presented in Table 2.

**Table 2:** Statistical results

	*n*	Mean	Std. deviation	Min	Max
RECIST analysis					
Partial response	11 (64.7%)				
Stable disease	5 (29.4%)				
Progressive disease	1 (5.9%)				
Complete response (CR)	0 (0%)				
No target lesion	2				
CA125 (IU/l) levels					
Pre-NACT	23	2,725.49	7,292.59	171.1	35,757.0
Post-NACT	22	105.27	146.05	9.9	548.0
Reduction (%)		85.66%	28.99%	− 40.83%*	98.81%
PCI					
Pre-NACT	23	29.91	4,49	25	39
Post-NACT	23	12.65	10.17	0	29
Reduction		17.26	10.31	2	39

^*^Negative reduction increase of CA125 level

### RECIST analysis

According to the RECIST 1.1 guidelines [3], 11 patients (67.7%) showed partial response (PR), 5 (29.4%) were categorized as stable disease (SD), and 1 (5.9%) was classified as progressive disease (PD) after NATC. In the case of two patients, the RECIST analysis could not be carried out, because no target lesions could be identified.

In 81.8% of the patients with PR, complete tumor resection was achieved during IDS after NACT. The one patient showing PD according to RECIST criteria showed a reduction of the tumormarker by 81%. Therefore, the patient was still considered to undergo IDS. During IDS, a reduction of the tumor burden was noticed. However, this patient could not undergo CCR due to irresectable liver metastasis at the hilium. 80% of those classified as SD underwent IDS with CCR. Intraoperatively, all of them showed a reduction of the tumor burden, regardless of the radiologic analysis.

### CA-125

**Fig. 2:**
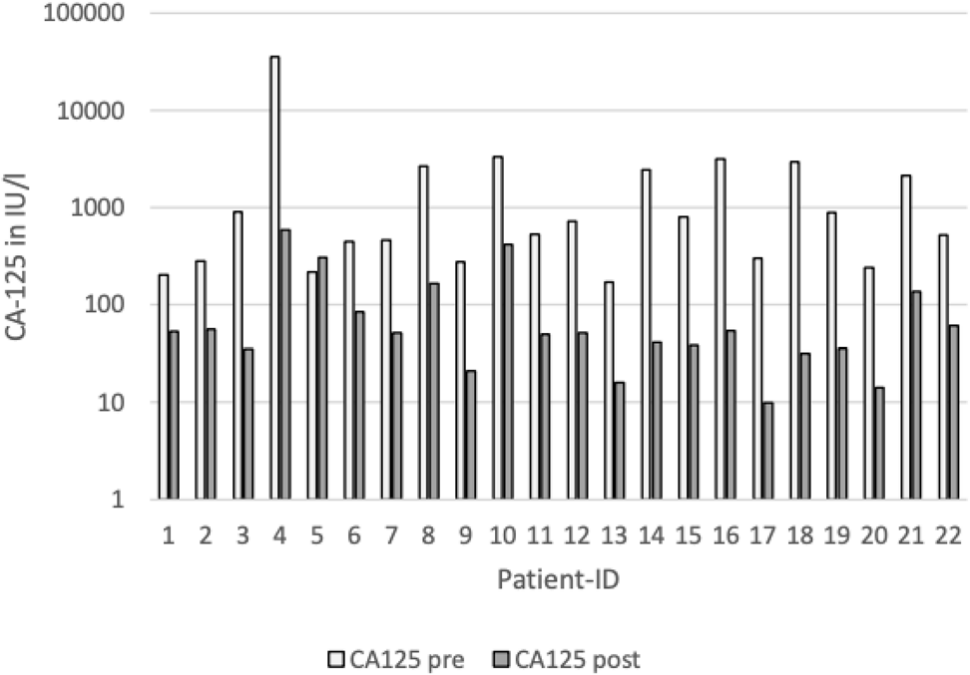
Patient’s level of tumor marker CA-125 before and after neoadjuvant CTX (logarithmic scale)

CA-125 values at baseline staging ranged from 171.0 IU/l to 35,737.0 IU/l. The CA-125 levels showed a decrease after NACT in all but one patient (patient-ID 5, see Fig. 2) after NACT.

After neoadjuvant treatment, CA-125 levels ranged from 9.90 IU/l to 584.0 IU/l. The CA-125 levels decreased by a mean of 85.66%. For those patients where CCR was reached, the mean reduction was 83.76%, for those where CCR was not achieved, the mean reduction was 90.74%. CCR was achieved in patient-ID 5, with elevated CA-125 levels after NACT, since the tumor burden showed to be reduced during IDS.

In a binary logistic regression analysis, no significant association could be found in respect to relative CA-125 reduction and independent predictable variable CCR (*p* = 0.642).

### PCI

**Fig. 3:**
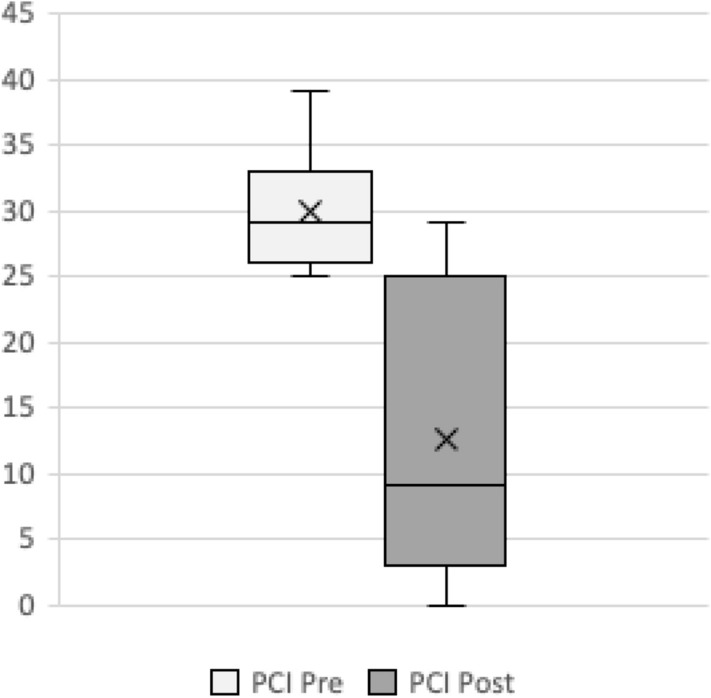
PCI score before and after neoadjuvant CTX

The mean PCI before NATC was 29.91, ranging from 25 to 39. After neoadjuvant treatment, the mean PCI was 12.65 with a range from 0 to 29 (Fig. 3). The PCI reduction ranged from a minimum of 2 points to complete reduction of all 39 points in one patient. Those patients where CCR was achieved had a mean PCI reduction of 20.24 points, while those where CCR could not be performed had a significantly lower mean PCI reduction of 8.83 points. We saw a significant association in the binary logistic regression between the extent of the PCI reduction and complete cytoreduction [OR = 1.17 (95% CI 1.01; 1.35), *p = *0.035]. Irrespectively of the different initial PCI score, there was a significant correlation between the extent of the PCI reduction and complete cytoreduction [OR = 1.17 (95% CI 1.01; 1.36), *p = *0.035].

### ROC curves

**Fig. 4:**
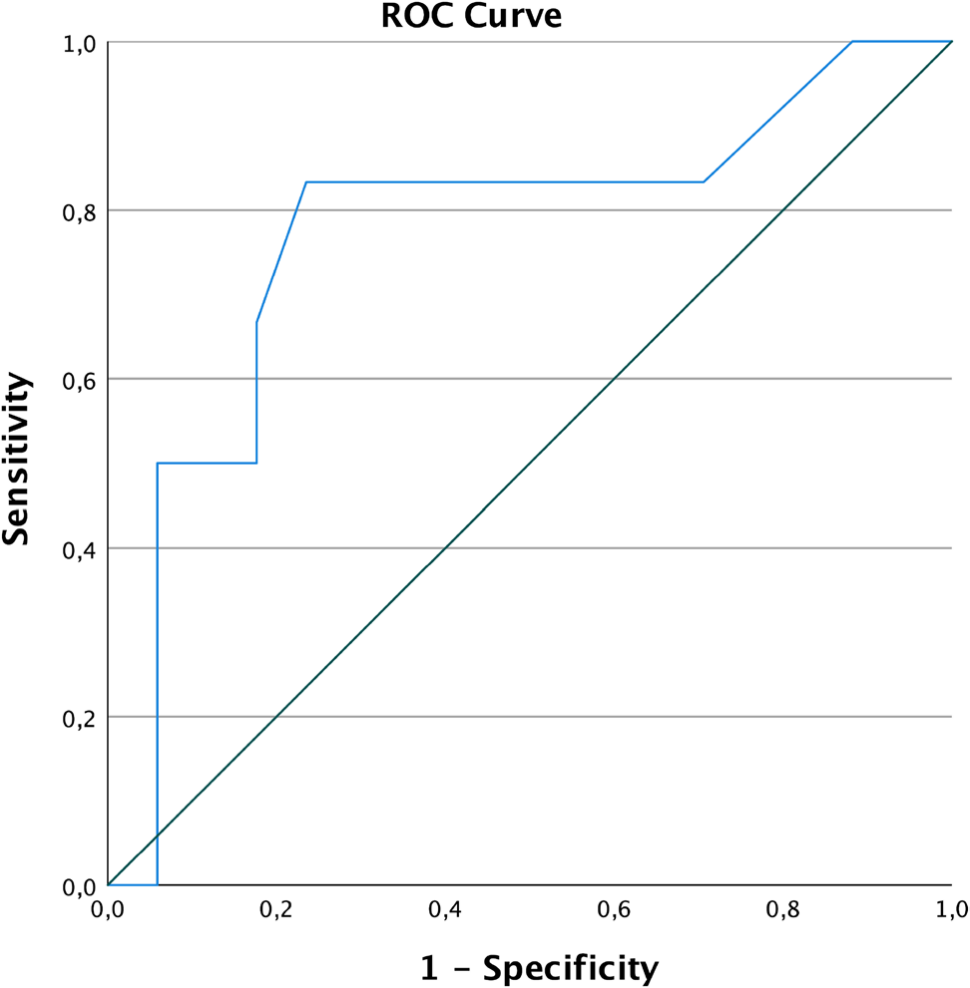
ROC PCI after NACT no complete cytoreduction (non-CCR)

To find the best possible value of the PCI score to predict CCR, ROC curves were used. The predictive quality of the PCI score for non-CCR after NACT is shown in the ROC curve in Fig. 4. In our collective, the AUC is 0.77 [(95% CI 0.53–1.00), *p = *0.050]. The anticipated cut-off at a PCI of < 25 had a sensitivity of 82.4% and a specificity of only 50% (Youden’s Index = 0.32). In our cohort, the “optimal” cut-off value according to Youden’s Index[29] for the PCI would be < 17 with a sensitivity of 77% and a specificity of 83% (Youden’s Index = 0.60).

**Fig. 5:**
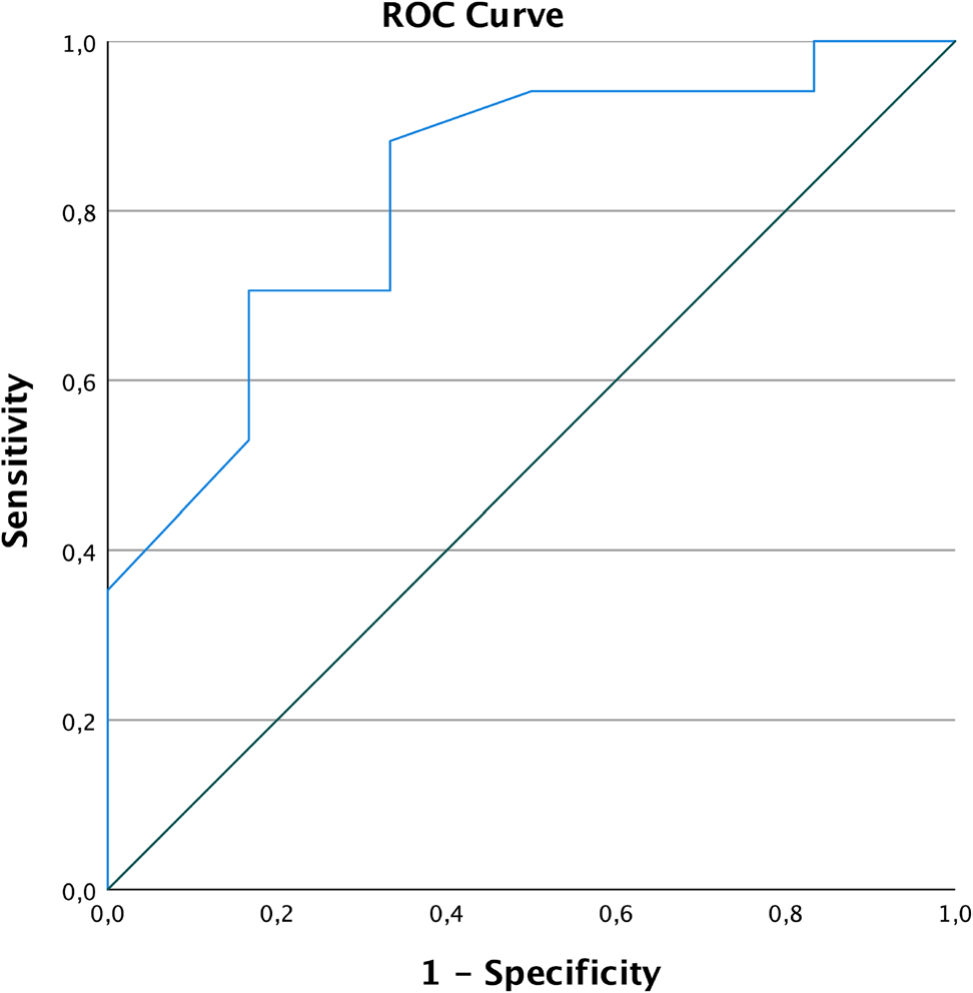
ROC PCI reduction complete cytoreduction (CCR)

In a further analysis, we statistically tried to improve specificity and sensitivity by looking at the (absolute) PCI reduction (Fig. 5), rather than just the PCI score after NATC. Here, the AUC is 0.82 [(95% CI 0.63–1.00), *p = *0.021]. The optimal cut-off value according to Youden’s Index is a PCI reduction of > 8.5 points, where the sensitivity was 88% and the specificity was 67% (Youden’s Index 0.55).

In particular, both sensitivity and specificity of a PCI reduction > 8.5 points were higher than for a PCI score < 25 after NACT.

### Correlations

There was no significant correlation between the PCI reduction and the CA-125 reduction (%) (r = − 0.21, *p = *0.348) regarding CCR. Comparing the RECIST results and PCI scores, no correlations were identified. One patient with PD according to RECIST had a reduction of 14 PCI points, which is close to the mean PCI reduction. Those patients who were classified PR and SD showed a mean reduction of 16.91 and 18.20 points, respectively.

We observed similar results when comparing RECIST and CA-125 reduction. Patients with PR according to RECIST had a mean reduction of CA-125 by 78%, but included the patient with elevated CA-125 levels after treatment (increase by 40.83%). The patient with PD showed a CA-125 reduction of 81.17%.

## Discussion

### Summary of main results

Patients will only benefit from surgery regarding overall survival (OS) and progression-free survival (PFS) when CCR can be achieved during IDS. Therefore, careful patient selection is needed. In recurrent ovarian cancer, the AGO-Score [1] has been developed to objectify patient selection criteria to predict surgical outcome. The prospective AGO-DESKTOP III gives evidence that surgery for recurrent cases seems to be of benefit for selected patients undergoing complete resection [1, 30]. Fagotti [31] suggested an alternative scoring system for primary ovarian cancer to predict operability to Sugarbakers PCI [2] using laparoscopy. However, there is no prognostic tool in the neoadjuvant setting of primary ovarian cancer or score to help selecting those patients. To distinguish patients that will benefit from IDS regarding OS and PFS from those who will not, we need scores and reliable predictive markers to help with decision-making.

### Results in the context of published literature

Kessous et al. [19] suggest a normal Serum CA125 lower than 30 U/ml to predict surgical outcome. Rodriguez et al.[20] found that patients with a preoperative CA-125 of ≤ 100 U/mL were highly likely to be cytoreduced to no residual disease. Vasudev et al. [21] analyzed the CA125 regression during NACT and found it to be strongly predictive of optimal cytoreduction, but there was no clear cut-off value defined that is efficient in every day practice. Our data showed that levels of CA-125 did not correlate with the tumor burden, neither at time of diagnosis nor after NACT. The levels ranged from 171 IU/l to 35,737 IU/l at time of diagnosis in our patients. In contrast to Kessous [19] or Rodriguez [20] data, we were able to achieve CCR in patients with much higher CA-125 levels. Therefore, the cut-off value of 30 or 100 U/ml as suggested does not seem to be an ideal cut-off value for the prediction of CCR.

We could also not reproduce the data of Vasudev [21]. According to our data, reduction in CA-125 levels failed to prove as a significant predictor to achieve CCR during IDS. Therefore, we could not reproduce the data [19–21]. Absolute CA -125 levels or reduction alone should not be used as a single predictor for CCR and therapeutic decision-making.

Even though the statistical analysis of the RECIST CT-scan results showed a trend that suggests predictive power, it was not significant (*p = *0.931). We could not confirm the data of Bregar et al. [24], stating that changes seen in CT scans before and after neoadjuvant chemotherapy could minimize reader variability using RECIST and predict surgical outcome. It is important to keep in mind that the used target lesions [3] are usually not the lesions responsible for leaving macroscopic tumor burden. Especially, the small but extensive peritoneal carcinomatosis (< 5 mm) does not show in the CT-scan and, therefore, cannot be assessed radiologically [32], but is often the limiting factor for CCR [10]. Furthermore, patients had to be excluded from the RECIST analysis, because no target lesions could be identified according to the RECIST criteria. Therefore, based on our study population, RECIST criteria alone should not be used to predict CCR and are not well applicable to patients with OC.

The PCI score < 25 at the time of diagnosis is a good prognostic tool to predict CCR in ovarian cancer at primary debulking according to Kroll et al. [2]. In IDS, we demonstrated a significant predictive value for the PCI for CCR after NACT. Regardless of the PCI baseline values, the reduction of the PCI showed to be a powerful predictor for CCR. Comparing the ROC curves, the PCI reduction proved to be a better prognostic factor than the absolute PCI values (Figs. 4 and 5). Using a cut-off of 17 in the neoadjuvant setting was the most sensitive and specific marker for CCR.

According to our data, the anticipated cut-off of PCI < 25 suggested to be a predictor for CCR, but was not significant. The cut-off to achieve CCR is different in PDS and IDS (17 vs. 25).

This indicates that the PCI must be assessed differently in neoadjuvant setting than in a primary situation. Even if the PCI is numerically identical, the extent of the disease may differ. It is not necessarily surprising that the PCI cut-off for CRR after NACT is lower than in the primary setting. Some tumor masses will stay non-resectable due to their location. Furthermore, it is important to keep in mind that patients who underwent NACT are usually in a worse general state of health than patients undergoing primary surgical debulking. Therefore, the surgical radicality might be constrained. It should be noted that the PCI can only be evaluated intraoperatively and thus means an intervention for the patient.

### Implications for practice and future research

In recurrent ovarian cancer, Harter et al. showed that peritoneal carcinomatosis was a negative predictor for complete resection, but had no effect on the prognosis if complete resection could be achieved [33]. Neither CA125 levels nor radiologic imaging alone is helpful to predict surgical outcome in patients after NACT. The reduction of the PCI score (before and after neoadjuvant chemotherapy) seems to be a better prognostic factor for CCR. Whether the reduction in the PCI after NACT itself has an influence on the OS and PFS in the primary setting cannot yet be assessed based on the current data situation. For this purpose, larger data collections are necessary over a longer observation period. We are aware that these assumptions are based on a small patient cohort and larger analyses, preferably with data from multiple centers, is required to strengthen our results.

## Conclusions

It can be stated that the PCI score cannot only be used to predict operability at the initial diagnosis, as shown by Kroll et al. [2], but also to evaluate the effect of NACT on EOC and to predict surgical outcome at the time of IDS. According to our small cohort, the “optimal” cut-off value in the sense of the highest possible sensitivity and specificity for the PCI after NACT would be 17. Of course, surgeons will always use several parameters to decide in operability, such as the general status and the location of the tumor. However, the PCI score could be a useful scoring system to objectify and compare the extend of the disease and predict surgical outcome at the time of IDS.

## References

[1] Harter P, Sehouli J, Vergote I (2021). Randomized trial of cytoreductive surgery for relapsed ovarian cancer. N Engl J Med.

[2] Lampe B, Kroll N, Piso P, Forner DM, Mallmann P (2015). Prognostic significance of sugarbaker’s peritoneal cancer index for the operability of ovarian carcinoma. Int J Gynecol Cancer.

[3] Bogani G, Matteucci L, Tamberi S (2019). RECIST 1.1 criteria predict recurrence-free survival in advanced ovarian cancer submitted to neoadjuvant chemotherapy. Eur J Obstet Gynecol Reprod Biol.

[4] Torre LA, Trabert B, DeSantis CE (2018). Ovarian cancer statistics, 2018. CA Cancer J Clin.

[5] Leitlinienprogramm Onkologie. S3-Leitlinie Diagnostik, Therapie und Nachsorge maligner Ovarialtumoren, Langversion 4.0, AWMF-Registriernummer: 032/035OL. 2020:1–153. https://www.leitlinienprogramm-onkologie.de/fileadmin/user_upload/Downloads/Leitlinien/Ovarialkarzinom/Version_4/LL_Ovarialkarzinom_Langversion_4.0.pdf.

[6] du Bois A, Reuss A, Pujade-Lauraine E, Harter P, Ray-Coquard I, Pfisterer J (2009). Role of surgical outcome as prognostic factor in advanced epithelial ovarian cancer: a combined exploratory analysis of 3 prospectively randomized phase 3 multicenter trials: by the Arbeitsgemeinschaft Gynaekologische Onkologie Studiengruppe Ovarialkarzin. Cancer.

[7] Aletti GD, Gostout BS, Podratz KC, Cliby WA (2006). Ovarian cancer surgical resectability: relative impact of disease, patient status, and surgeon. Gynecol Oncol.

[8] Shih KK, Chi DS (2010). Maximal cytoreductive effort in epithelial ovarian cancer surgery. J Gynecol Oncol.

[9] Elattar A, Bryant A, Winter-Roach BA, Hatem M, Naik R (2011). Optimal primary surgical treatment for advanced epithelial ovarian cancer. Cochrane database Syst Rev.

[10] Fagotti A, Gallotta V, Romano F (2010). Peritoneal carcinosis of ovarian origin. World J Gastrointest Oncol.

[11] Querleu D, Planchamp F, Chiva L (2017). European society of Gynaecological Oncology (ESGO) guidelines for ovarian cancer surgery. Int J Gynecol Cancer.

[12] Jacquet P, Sugarbaker P (1996). Current methodologics for clinical assesment of patients with peritoneal carcinomatosis. J Exp Clin Cancer Res.

[13] Angeles MA, Migliorelli F, Del M (2021). Concordance of laparoscopic and laparotomic peritoneal cancer index using a two-step surgical protocol to select patients for cytoreductive surgery in advanced ovarian cancer. Arch Gynecol Obstet.

[14] Wright AA, Bohlke K, Armstrong DK (2016). Neoadjuvant chemotherapy for newly diagnosed, advanced ovarian cancer: Society of Gynecologic Oncology and American Society of Clinical Oncology Clinical Practice Guideline. J Clin Oncol.

[15] Vergote I, Coens C, Nankivell M (2018). Neoadjuvant chemotherapy versus debulking surgery in advanced tubo-ovarian cancers: pooled analysis of individual patient data from the EORTC 55971 and CHORUS trials. Lancet Oncol.

[16] du Bois A, Quinn M, Thigpen T (2005). 2004 consensus statements on the management of ovarian cancer: final document of the 3rd International Gynecologic Cancer Intergroup Ovarian Cancer Consensus Conference (GCIG OCCC 2004). Ann Oncol Off J Eur Soc Med Oncol.

[17] Kehoe S, Hook J, Nankivell M (2015). Primary chemotherapy versus primary surgery for newly diagnosed advanced ovarian cancer (CHORUS): an open-label, randomised, controlled, non-inferiority trial. Lancet.

[18] Vallius T, Hynninen J, Auranen A (2014). Serum HE4 and CA125 as predictors of response and outcome during neoadjuvant chemotherapy of advanced high-grade serous ovarian cancer. Tumour Biol.

[19] Kessous R, Wissing MD, Piedimonte S (2020). CA-125 reduction during neoadjuvant chemotherapy is associated with success of cytoreductive surgery and outcome of patients with advanced high-grade ovarian cancer. Acta Obstet Gynecol Scand.

[20] Rodriguez N, Rauh-Hain JA, Shoni M (2012). Changes in serum CA-125 can predict optimal cytoreduction to no gross residual disease in patients with advanced stage ovarian cancer treated with neoadjuvant chemotherapy. Gynecol Oncol.

[21] Vasudev NS, Trigonis I, Cairns DA (2011). The prognostic and predictive value of CA-125 regression during neoadjuvant chemotherapy for advanced ovarian or primary peritoneal carcinoma. Arch Gynecol Obstet.

[22] Le T, Faught W, Hopkins L, Fung-Kee-Fung M (2008). Importance of CA125 normalization during neoadjuvant chemotherapy followed by planned delayed surgical debulking in patients with epithelial ovarian cancer. J Obstet Gynaecol Can.

[23] Ramspott JP, Baert T, MacKintosh ML (2021). Response evaluation after neoadjuvant therapy: evaluation of chemotherapy response score and serological and/or radiological assessment of response in ovarian cancer patients. Arch Gynecol Obstet.

[24] Bregar A, Mojtahed A, Kilcoyne A (2019). CT prediction of surgical outcome in patients with advanced epithelial ovarian carcinoma undergoing neoadjuvant chemotherapy. Gynecol Oncol.

[25] Asp M, Malander S, Wallengren N-O (2022). The role of computed tomography in the assessment of tumour extent and the risk of residual disease after upfront surgery in advanced ovarian cancer (AOC). Arch Gynecol Obstet.

[26] Yoneoka Y, Ishikawa M, Uehara T (2019). Treatment strategies for patients with advanced ovarian cancer undergoing neoadjuvant chemotherapy: interval debulking surgery or additional chemotherapy?. J Gynecol Oncol.

[27] Bhatt A, Rousset P, Benzerdjeb N (2021). Clinical and radiologic predictors of a pathologic complete response to neoadjuvant chemotherapy (NACT) in patients undergoing cytoreductive surgery for colorectal peritoneal metastases: results of a prospective multi-center study. Ann Surg Oncol.

[28] Eisenhauer EA, Therasse P, Bogaerts J (2009). New response evaluation criteria in solid tumours: revised RECIST guideline (version 1.1). Eur J Cancer.

[29] Youden WJ (1950). Index for rating diagnostic tests. Cancer.

[30] Pignata S, C Cecere S, Du Bois A, Harter P, Heitz F (2017). Treatment of recurrent ovarian cancer. Ann Oncol Off J Eur Soc Med Oncol.

[31] Fagotti A, Ferrandina G, Fanfani F (2006). A laparoscopy-based score to predict surgical outcome in patients with advanced ovarian carcinoma: a pilot study. Ann Surg Oncol.

[32] Diop AD, Fontarensky M, Montoriol P-F, Da Ines D (2014). CT imaging of peritoneal carcinomatosis and its mimics. Diagn Interv Imaging.

[33] Harter P, Hahmann M, Lueck HJ (2009). Surgery for recurrent ovarian cancer: role of peritoneal carcinomatosis: exploratory analysis of the DESKTOP I trial about risk factors, surgical implications, and prognostic value of peritoneal carcinomatosis. Ann Surg Oncol.

